# Dysregulated wound healing in the pathogenesis of urogynecologic mesh complications

**DOI:** 10.1038/s41598-023-48388-8

**Published:** 2023-12-05

**Authors:** Amanda M. Artsen, Rui Liang, Leslie Meyn, Megan S. Bradley, Pamela A. Moalli

**Affiliations:** Department of Obstetrics, Gynecology and Reproductive Sciences at Magee Womens Hospital, Magee-Womens Research Institute, University of Pittsburgh, 204 Craft Avenue 312A, Lab A320, Pittsburgh, PA 15213 USA

**Keywords:** Translational research, Urogenital reproductive disorders, Implants

## Abstract

To test the hypothesis that dysregulated wound healing is associated with Urogynecologic mesh complications, we collected vaginal cell secretions using vaginal swabs after polypropylene mesh implantation in patients with (N = 39) and without (N = 40) complication. A customized multiplex immunoassay measured markers of inflammation (MCP-1, IGFBP-1, IL-2, IL-10, IL-17, PDGF-BB, bFGF, IL-1b, IL-6, IL-12p70, TNF-α), neuroinflammation (IL-1RA, TGF-β, IL-15, IL-18, IL-3, M-CSF), angiogenesis (VEGF), and matrix proteins (fibronectin, tenasin c, thrombospondin-2, lumican) between groups. Patients with complications were younger, heavier, implanted with mesh longer, and more likely to be ever smokers. A 5 kg/m^2^ BMI increase and ever-smoking were associated with a 2.4-fold and sixfold increased risk of complication, respectively. Patients with the highest tertile of bFGF, fibronectin, thrombospondin-2, TNF-β, or VEGF had an odds ratio (OR) of 11.8 for having a mesh complication while ≥ 3 elevated had an OR of 237 while controlling for age, BMI, and smoking. The highest tertile of bFGF, thrombospondin-2, and fibronectin together perfectly indicated a complication (*P* < 0.0001). A receiver-operator curve for high bFGF, thrombospondin-2, and fibronectin showed excellent discrimination between complications and controls (AUC 0.87). These data provide evidence of dysregulated wound healing in mesh complications. Modifiable factors provide potential targets for patient counseling and interventions.

## Introduction

Pathologic fibrosis surrounding biomedical devices has long hindered the use of a variety of implants, including breast implants, insulin pumps, artificial joints, and surgical meshes^1–3^. The foreign body response, which in a well-integrated implant begins with inflammation and reaches homeostasis with a thin fibrotic capsule surrounding the implant, is a normal and expected tissue response^4^. However, complications can arise when the host tissue repair pathway after foreign body is implanted becomes dysregulated. These responses, particularly the development and control of fibrosis, are highly organ specific^5^.

Urogynecologic surgeons have used polypropylene meshes for decades to improve the durability of prolapse repairs and reduce invasiveness of surgery for incontinence, with more than 100,000 patients having prolapse mesh implanted annually^6–8^. However, even lightweight polypropylene meshes are associated with fibrosis-related pain and exposure, a condition in which the mesh shows through the vaginal epithelium in 4–10% of cases^9–11^. This can cause bleeding, dyspareunia for the patient and partner, and other complications^12^.

Healing after mesh implantation is complex and not only stimulates the foreign body response but also requires tissue ingrowth into the pores of the mesh. Like the acute foreign body response, the first stage of wound healing is a pro-inflammatory hemostatic response that seals the wound and prevents infection. The second stage is tissue replacement, a phase that drives fibroblast activation and differentiation into myofibroblasts via increased TGF-β. Myofibroblasts secrete collagen providing a provisional matrix that covers the wound and places traction on the wound edges. In the third and final phase known as the resolving phase, inflammation subsides and collagen maturation is completed. During this phase, myofibroblasts disappear from the wound in a wave of apoptosis. When wound healing becomes dysregulated, both chronic wound and scarring states ensue from the inability of the wound to enter a resolving phase^13,14^. Stark parallels exist between prolonged inflammation and high extracellular matrix turnover seen in mesh exposure complications and chronic skin wounds suggesting that mesh exposure may result from arrest in the inflammatory phase of wound healing^13,15–25^.

Likewise, the events observed in polypropylene mesh pain complications parallel dermal scarring. Increasing histologic fibrosis surrounding polypropylene vaginal mesh explants has been linearly associated with higher pelvic pain visual analog scores in women who had mesh removed for a mesh-related complication^19^. In addition, TGF-β, a profibrotic cytokine in many organ systems, was doubled in women who had persistent pain after mesh removal compared to those who improved after removal^19^. Despite the critical role of the extracellular matrix in wound healing and the promise it holds for therapeutic intervention^26–28^, very little is known about this process in vaginal biomaterial integration including mesh.

We hypothesized that patients with mesh complications will have evidence of abnormal wound healing. We further posited that proteins indicative of dysregulated extracellular matrix remodeling would be elevated in mesh complications, but that inflammatory markers would be similar between groups. To test this hypothesis, we aimed to assess a wide range of soluble markers of inflammation, pain and neuroinflammation, angiogenesis, and a hypothesis-driven set of matrix proteins critical for transition to the resolving phase of wound healing in patients with and without mesh complications. Clinical and demographic variables were collected and compared between the groups to identify factors that increase risk. As an exploratory aim, the same markers were compared in patients who reported improvement in pain after mesh removal to those who reported no improvement.

## Methods

### Patient selection

This study was approved by the University of Pittsburgh Institutional Review Board. All study procedures were performed in accordance with the relevant guidelines and regulations including STROBE guidelines. All patients presenting to the Magee Women’s Center for Bladder and Pelvic Health at the University of Pittsburgh who were scheduled to undergo a complete vaginal mesh excision for the complications of vaginal mesh exposure or mesh-related pain were offered enrollment in the Mesh Biorepository Cohort Study. Informed consent was obtained from all participants. Patients with mesh exposure into the urethra, bladder or rectum or clinical signs of infection (fever, elevated white blood cell count) were excluded. Age, BMI, gravity, parity, smoking status, menopausal status, use and type of hormone replacement therapy, comorbidities, variables describing the initial mesh placement and other demographic variables were collected at the time of enrollment using a standardized form developed a priori. Former smokers and current smokers were grouped together as “ever smokers.” Vaginal swabs were used to collect cells and secretions from the vaginal apex at the time of mesh removal. Complete vaginal mesh excisions were performed as described in the AUGS/IUGA Joint Statement on the Management of Mesh-Related Complications for the FPMRS Specialist^12^. Symptom questionnaires including visual analog scales (VAS) for pelvic pain scores and PFD-20 questionnaires were completed at enrollment and 6 months after removal. To meet the necessary sample size (see calculation below), samples were randomly selected from this biorepository.

For the control group, all patients with prior mesh placement (prolapse or incontinence mesh) at our institution without subsequent complications who were seen in the clinic for routine follow up beginning in 5/2019 were offered enrollment as controls. During the enrollment period, of the 84 patients invited to participate, 5 declined. Vaginal swabs from control patients were collected in the same way during routine pelvic examination until the necessary sample size was achieved (see calculation below).

### Swab processing and pellet histology

After collection, swabs were placed into DPBS and frozen at − 80 degrees until all 79 samples were collected. Swabs were then thawed, vortexed, and centrifuged to remove cells and debris from the swabs. Slides were created from two test vaginal swabs prior to and after processing. These slides were stained with H&E to analyze cell type in the pellet and confirm removal of cells from the supernatant. Protein from the supernatant was then concentrated using centrifugation in commercial concentrator tubes (Thermo Scientific, Waltham, MA) until the protein concentration exceeded 2 mg/mL. Filtrate was confirmed to have an undetectable protein level indicating low protein loss.

### Analyte quantification

A customized bead-based multiplex immunoassay (Luminex, R&D Systems, Minneapolis MN) was used to measure markers of inflammation or fibrosis (MCP-1, IGFBP-1, IL-2, IL-10, IL-17, PDGF-BB, bFGF, IL-1 β, IL-6, IL-12p70, TNF-α), pain and neuroinflammation (IL-1RA, TNF-β, IL-15, IL-18, IL-3, M-CSF), angiogenesis (VEGF), and matrix proteins critical for transition to resolving phase (fibronectin, tenasin c, thrombospondin 2, and lumican). Due to incompatibility with other analytes in the bead-based assay, TGF-β on 20 mesh and 15 control samples that had sufficient protein was analyzed separately using ELISA. An internal control was run on each plate.

### Statistical analysis

Using previously published data on PDGF-BB^19^ from mesh explants and controls where 59% of mesh samples compared to 13% of controls were in the highest tertile with a corrected alpha of 0.0023 and a power of 90%, it was calculated that 38 samples per group (76 total) were needed to detect a difference. 79 total samples were collected to account for the potential for lost or insufficient samples and all of these were able to be used for analysis. Minimal plate variation was noted by comparing standard curves, however, to minimize plate-to-plate variation, data were normalized to an internal control that was run on all plates.

Demographic and clinical characteristics were compared between women with and without mesh complications using Student’s t-, Mann–Whitney U, and Fisher’s exact tests, where appropriate. Analytes below the detectable limit were replaced with lower limit of detection/2. Analytes were first analyzed as continuous variables and then significant variables were categorized into the highest tertile vs middle/low tertiles to assess the effect of high values of each analyte. The percentages of patients with the highest tertile of analyte were compared between groups using Fisher’s exact test with a Bonferroni correction (a_1_ = 0.0023). Logistic regression models containing analytes with *P* < 0.01 by univariate analysis assessed the odds of having a complication by analyte while controlling for age, BMI and smoking. Receiver-operator curves were generated for analytes that were different between groups. Fisher’s exact and Mann–Whitney U tests were performed to compare analytes in patients with complications who met the minimally important clinical difference of 13 mm improvement on a visual analog scale^29^ after removal (referred to as “responders”) as compared to those who did not. Receiver-operative curves were generated for these analytes.

## Results

Patients with mesh complications were on average 9 years younger (56.0 ± 11.9 vs. 65.0 ± 8.9), heavier (BMI = 29.9 ± 5.5 vs. 26.6 ± 3.9) and more often ever smokers (58% or 23/40 vs. 28% or 11/39) than those without mesh complications (*p* < 0.05, Table 1). 87% of patients in the study were postmenopausal. Patients with mesh complications were more likely to be on both systemic and vaginal estrogen therapy (*p* < 0.05). They were also more likely to have received a mid-urethral sling as compared to a prolapse mesh and have a longer duration of implantation (*p* < 0.05, Table 1). The groups had similar rates of diabetes diagnosis. Each 5 kg/m^2^ BMI increase and ever smoking were associated with a 2.4-fold (*p* = 0.041) and sixfold (*p* = 0.026) increased risk of complication, respectively. When accounting for these variables, age was no longer a significant predictor of mesh complication.

**Table 1:** Demographics of women with and without mesh complications from whom vaginal swabs were collected for analysis.

Variables	Mesh complication N = 40	No mesh complication N = 39	*P* value
Age (years)	56.0 ± 11.9	65.0 ± 8.9	0.001
BMI (kg/m^2^)	29.9 ± 5.5	26.6 ± 4.0	0.003
Current/Former smoker	23 (58%)	11 (28%)	0.012
Vaginal parity (median; range)	2 (0–5)	2 (0–6)	0.20
Hormone replacement therapy, any	24 (67%)	15 (38%)	0.021
Systemic estrogen	6 (15%)	1 (4%)	0.015
Vaginal estrogen	23 (58%)	14 (36%)	0.014
Premenopausal	8 (20%)	2 (5%)	0.087
Midurethral sling	24 (60%)	8 (21%)	0.001
Duration (years; median, IQR)	5.5 (3.2–8.7)	2.4 (1.3–7.0)	0.009
Diabetes	2 (5%)	2 (5%)	0.99
Chronic steroid use	3 (8%)	0 (0%)	0.24

Data are mean ± SD or n(%) unless otherwise specified. BMI, body mass index; IQR interquartile range.

**Figure 1:**
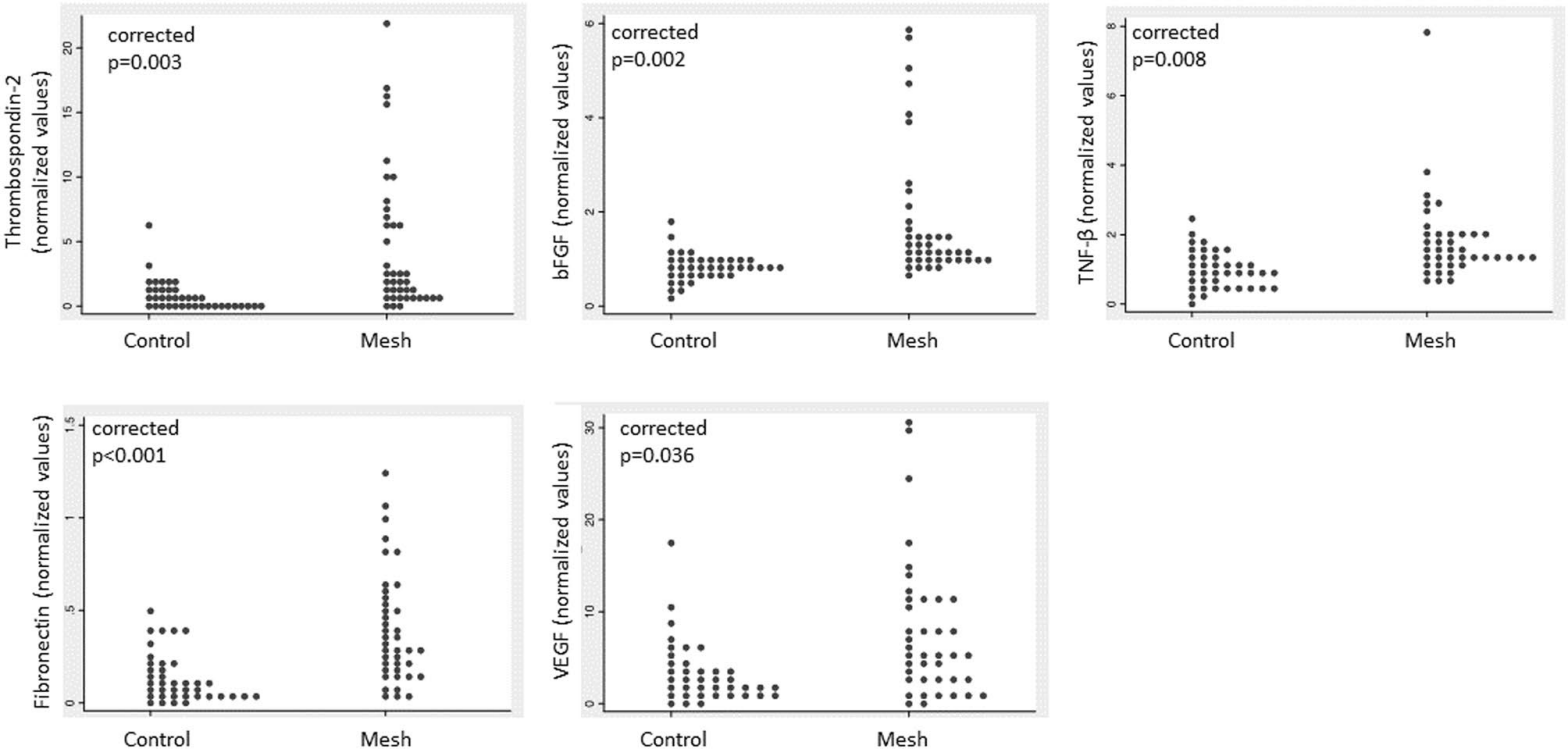
Comparison of 5 significant analytes in patients with mesh complications compared to controls normalized to an internal control to minimize plate variation. *P* values are Bonferroni-corrected.

**Figure 2:**
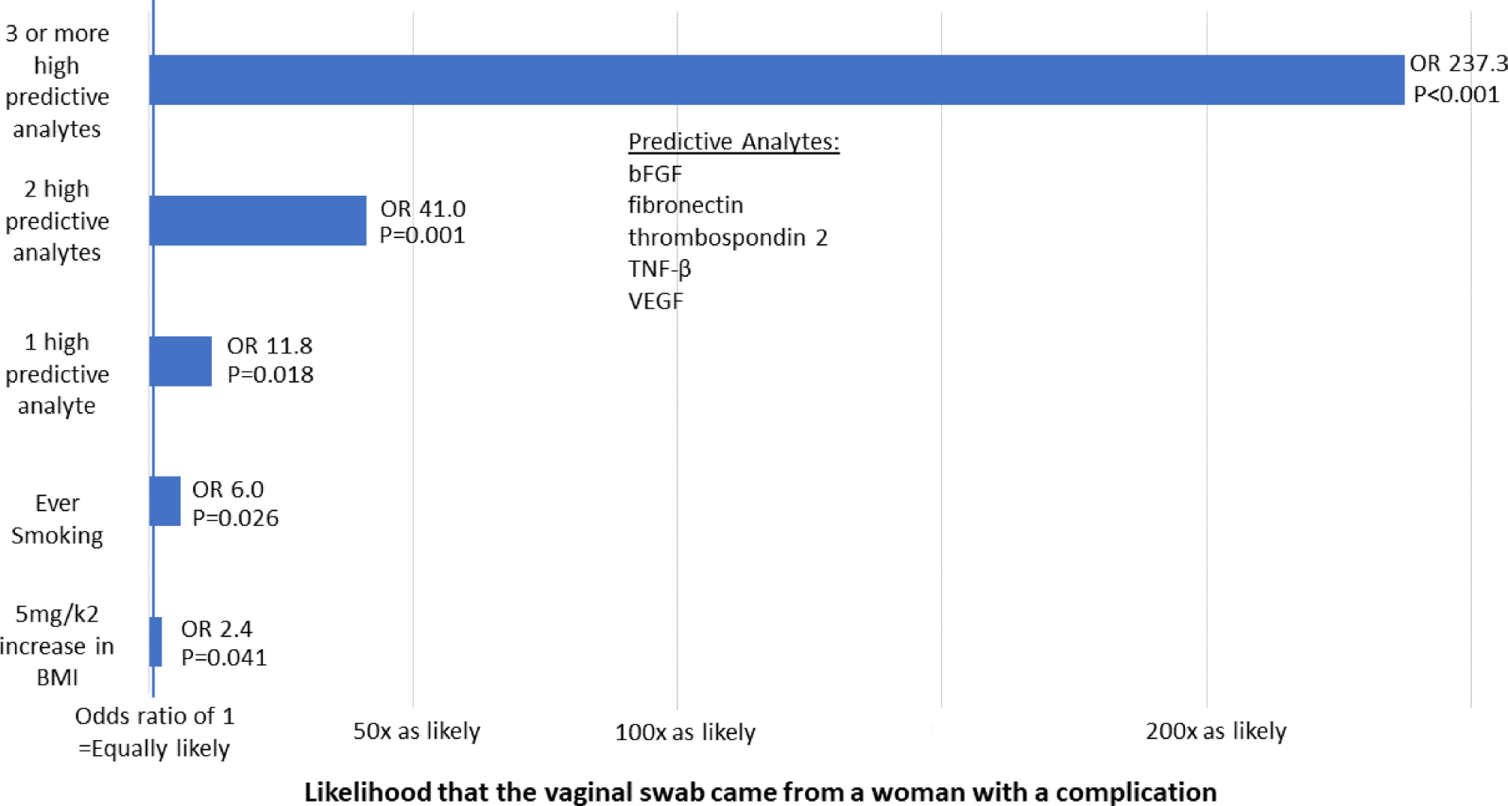
Predictors that the vaginal swab came from a patient with a complication.

Patients with mesh complications had higher bFGF, fibronectin, thombospondin-2, TNF-β and VEGF than patients with no complications (Fig. 1, corrected *p* value < 0.05). No other analytes were statistically different between groups. When these 5 analytes were grouped into tertiles, more patients with mesh complications had the highest tertile of bFGF, fibronectin, thombospondin-2 (Table 2, corrected *p* value < 0.05). There were no differences in these analytes between sling and prolapse mesh complications (*p* > 0.05). Logistic regression models controlling for age, BMI and smoking status, demonstrated that swabs with high levels of any one of these analytes: bFGF, fibronectin, thrombospondin 2, TNF-β or VEGF had an odds ratio (OR) of 11.8 of having come from a patient with a complication. These odds increased further when 2 or 3 or more analytes were elevated (Fig. 2). The combination of high bFGF, and fibronectin and thrombospondin 2 perfectly indicated a complication swab (*P* < 0.0001) with a positive predictive value (PPV) of 100% and negative predictive value of 57%. When only sling mesh complications were included in the model, the same variables indicated a complication swab except for BMI, which was not different between slings with complications and slings without complications. There were no differences in analytes between pain and exposure complications.

**Table 2:** Soluble factors of women with and without mesh complications from whom vaginal swabs were collected for analysis.

Analyte	Mesh complication N = 40	No mesh complication N = 39	*P* value	*P* value, bonferroni-corrected
High bFGF	23 (57.5%)	4 (10.3%)	< 0.001	< 0.001
High fibronectin	20 (50.0%)	6 (15.4%)	0.002	0.036
High thrombospondin-2	23 (57.5%)	3 (7.7%)	< 0.001	< 0.001
High TNF-β	19 (47.5%)	7 (17.9%)	0.008	0.176
High VEGF	19 (47.5%)	7 (17.9%)	0.008	0.176

*bFGF* basic fibroblast growth factor, *TNF-β* tumor necrosis factor β, *VEGF* vascular endothelial growth factor. High indicates 3rd tertile.

**Figure 3:**
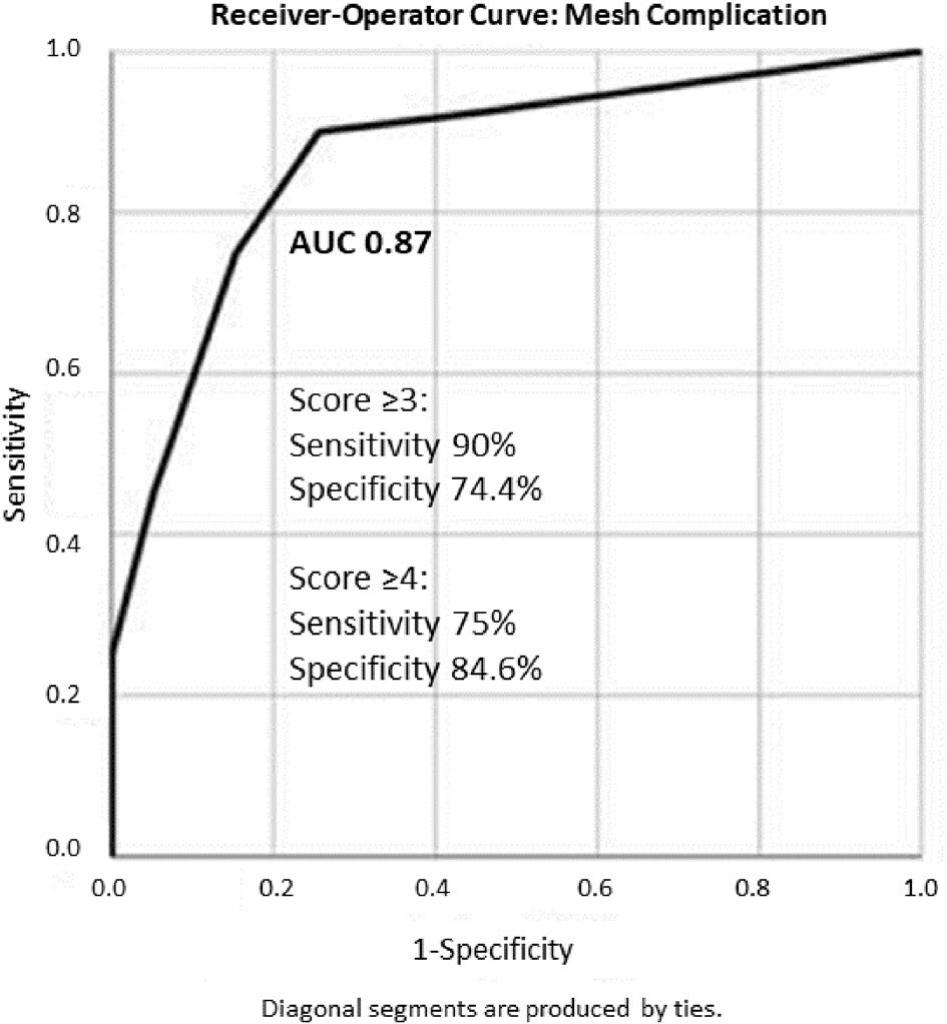
Receiver-operator curve showing characteristics predictive that the swab came from a patient with a complication of high bFGF, high fibronectin and high thrombospondin 2 for mesh complications. The curve represents a score computed by assigning points to the tertile of each analyte of bFGF, thrombospondin 2, and fibronectin. The first tertile is 0 points, 2nd is 1 point and 3rd tertile is two points. So for example, a score of 0 indicates first tertile for all three analytes, a score of 5 indicates 3rd tertile of one analyte or 2nd tertile or two analytes while a score of 6 represents 3rd tertile for all three analytes. A score of 4 or 5 (which were equal) optimized sensitivity/specificity.

A receiver-operator curve combining tertiles for bFGF, thrombospondin 2, and fibronectin showed excellent discrimination between complications and controls with an area under the curve of 0.87 (*p* < 0.001, Fig. 3).

**Figure 4:**
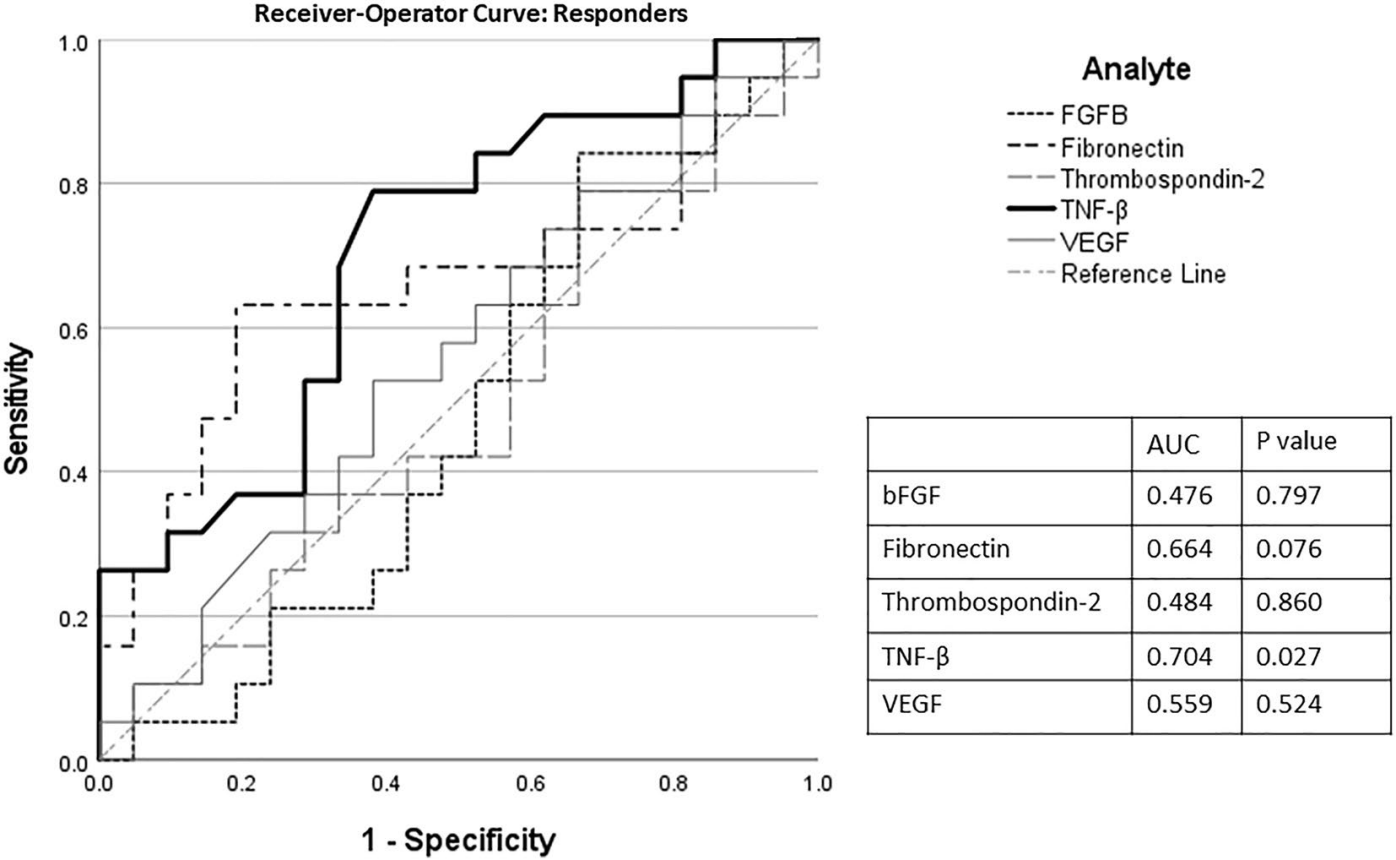
Receiver-operator curve showing characteristics predictive that the swab came from a patient with a complication of highest tertile of bFGF, fibronectin, thrombospondin-2, TNF-β, and VEGF for improvement in pain after removal.

On our exploratory analysis of individuals who marked at least a 13 mm improvement on the pelvic pain VAS score after mesh removal (responders) compared to those who did not (non-responders) showed that TNF-β was lower in responders 1.28 (IQR 1.12–1.64; normalized values) compared to non-responders 1.64 (IQR 1.44–2.07; normalized values*; P* = 0.018). A receiver-operator curve to predict nonresponse based on TNF-β demonstrated an area under the curve of 0.70 (*p* = 0.027, Fig. 4). There were no differences in any other analyte studied between these two groups. Finally, there were no differences in analytes between patients with exposure complications (32/40, 80%) and those with pain in the absence of exposure (8/40, 20%).

## Discussion

In comparing soluble factors promoting inflammation, fibrosis, pain, or wound healing in vaginal swabs obtained from patients with Urogynecologic polypropylene mesh complications versus those with mesh implanted without complications, our most important findings were that patients with mesh complications were more likely to have high bFGF, fibronectin, thrombospondin 2, VEGF and TNF-β in vaginal secretions obtained in vaginal swabs at the time of mesh removal and these analytes were highly indicative of a complication compared to controls, which suggests an inability to switch to a mature, well-organized matrix related to the pathophysiology of the complication or an underlying disorder. In addition, we observed elevated TNF-β in patients who experienced poor improvement in pain after mesh removal and that patients with a history of smoking or increased BMI were associated with an increased odds of a complication.

Fibronectin and thrombospondin-2 are extracellular matrix proteins that regulate extracellular matrix homeostasis, and excess levels can contribute to fibrosis^30,31^. Specifically, fibronectin is increased in glomerular and interstitial fibrosis^31,32^ and thrombospondin-2 has recently been identified as a possible target for decreasing fibrosis following regenerative cardiac cell grafts^33^ as well as a biomarker for severe liver fibrosis^34,35^. Because the ECM is already altered in patients with prolapse^36^, this may represent a double-hit phenomenon for prolapse patients, where an already disorganized ECM with decreased mature type I collagen and increased type III collagen is unable to terminate the wound healing response when exposed to mesh. Further research into the subtypes and source (plasma or cellular) of fibronectin involved in mesh complications and its interactions with the ECM may prove a useful future direction.

VEGF and bFGF are signal proteins that act synergistically to induce new blood vessel formation after injury^37^. Clinically, neovascularization has also been reported within the mesh capsule during surgical removal that increases risk of bleeding^38^. New blood vessel formation accompanies fibroproliferation in wound healing and is an important contributor to fibrosis in some diseases, where fragile new blood vessels can break and contribute to repetitive injury and inflammation^39,40^ PDGF-BB, previously shown to be elevated in mesh complications^19^, and both PDGF-BB and TGF-β are upstream promoters of angiogenesis by activation of the STAT3 pathway, which upregulates VEGF and bFGF. Alternatively, VEGF and bFGF can be released directly from the ECM when it is degraded, as is seen in mesh exposure complications. bFGF is also mitogenic for fibroblasts as well as vascular endothelial cells^41^. Interestingly, in our prior work looking at tissue samples of patients with mesh complications compared to vaginal biopsy controls, bFGF was decreased which could be due to reciprocal inhibitory feedback with PDGF-BB and TGF-β^19^. Here elevated bFGF could indicate a broader profibrotic environment in the vagina which was detected in vaginal swabs compared to a very local downregulation in the tissue immediately adjacent to the mesh complication.

Proinflammatory TNF is produced by activated macrophages (TNF-α) and lymphocytes (TNF-β; conflicting data for TNF-α)^42^ and its receptors mediate pain sensitivity, likely through increased neuronal firing and increased inflammation^43–45^. Increased levels of TNF-α has been demonstrated in fibromyalgia^46^, mouse models of arthritis^47^, and centralization of pain^48^. Like TNF-α, TNF-β binds to both TNF receptors but may have different cytotoxicity and binding affinities^42,49^. TNF-β, but not TNF-α, has been shown to be elevated in vulvodynia^50^. The elevation of TNF-β in patients who did not respond to removal suggest neuropathic pain as a mechanism of persistent pain after mesh removal.

Demographic differences between groups such as age and duration of implantation may be affected by those who present for follow up and were therefore available as controls. Current smoking is an accepted risk factor for mesh complications^51–53^. There is conflicting data on obesity as a risk factor for mesh complications^54,55^, however, like smoking, obesity results in increased reactive oxygen species and inflammation, which may tip the organ system into dysregulated healing when a foreign body is introduced. Obesity also increases levels of circulating estrogens made in fat^56^ which likely softens the tissue and increases the stress mismatch with the highly stiff implant. Because midurethral slings are substantially stiffer than prolapse meshes this mismatch can also be greater with slings, which may account for our finding that more women in the complication group had midurethral slings. This mismatch may also be more pronounced in young women, in whom the vagina is softer, and who are at increased risk for complication^57,58^.

Injury, wound healing, and fibrosis progress differently in different organs such as the skin, heart, liver, and lungs. The closest analog to the vagina is often thought to be the skin or esophagus^59^, however the vagina is a unique organ with distinct structure, material properties, microbiome and inflammatory niche^58^. These data provide an important insight into dysregulated wound healing in mesh complications.

Strengths include a large study population and a novel control group. Tissue biopsy controls for mesh complications are difficult to obtain because women without complication generally do not have it removed and this has led to an inability to distinguish risk factors and pathophysiologic changes in some studies^60^. Therefore, using vaginal swabs from women with well-integrated mesh is novel and provides a unique look into mesh healing. In addition, we used a custom designed kit to test our hypothesis by looking specifically at regulatory ECM proteins important in wound healing in addition to traditional markers of inflammation. Our study has several notable limitations including that swabs collected at the time of mesh excision, as these were, do not provide prospective data to determine pre-implanation risk of mesh complication. In addition, mesh types may have a differential effect and we are unable to stratify by mesh type due to multiple products used in this patient population; however, the fact that the mesh products while variable in textile properties exhibit similar complications suggests that they likely share common pathophysiology. Although operative notes were reviewed when available, data on difficulty of insertion, vaginal atrophy, and immediate postoperative concerns such as hematoma were not available for most patients which can contribute to this multifactorial problem. Finally, cells and secretions on swabs may result in smaller levels of analytes although we have previously shown good concordance in protein levels between vaginal swabs and biopsies^61^.

## Conclusions

In conclusion, high vaginal secretion levels of bFGF, thrombospondin 2, fibronectin, VEGF and TNF-β are strongly associated with ongoing mesh complications, with 3 elevated analytes having the strongest association. These data provide evidence of dysregulated wound healing in mesh complications. Further studies are needed to determine whether this plays a mechanistic role.

## Data Availability

Data is available upon request from the corresponding author.
